# Use of focal radiotherapy boost for prostate cancer: radiation oncologists’ perspectives and perceived barriers to implementation

**DOI:** 10.1186/s13014-023-02375-5

**Published:** 2023-11-11

**Authors:** Allison Y. Zhong, Asona J. Lui, Matthew S. Katz, Alejandro Berlin, Sophia C. Kamran, Amar U. Kishan, Vedang Murthy, Himanshu Nagar, Daniel Seible, Bradley J. Stish, Alison C. Tree, Tyler M. Seibert

**Affiliations:** 1 Department of Radiation Medicine and Applied Sciences, University of California San Diego, La Jolla, CA USA; 2 Department of Radiation Medicine, Lowell General Hospital, Lowell, MA USA; 3 Radiation Medicine Program, Princess Margaret Cancer Centre, University of Toronto, Toronto, Canada; 4 Department of Radiation Oncology, Massachusetts General Hospital, Harvard Medical School, Boston, MA USA; 5 Departments of Radiation Oncology and Urology, UCLA, Los Angeles, CA USA; 6 ACTREC, Tata Memorial Centre, Homi Bhabha National Institute, Mumbai, India; 7 Weill Cornell Medicine, New York, NY USA; 8 Anchorage and Valley Radiation Therapy Centers, Anchorage, AK USA; 9 Department of Radiation Oncology, Mayo Clinic, Rochester, MN USA; 10 The Royal Marsden NHS Foundation Trust/The Institute of Cancer Research, London, UK; 11 Departments of Radiology and Bioengineering, University of California San Diego, La Jolla, CA USA

**Keywords:** Prostate cancer, Radiotherapy, Focal boost, Tumor boost

## Abstract

**Background:**

In a recent phase III randomized control trial, delivering a focal radiotherapy (RT) boost to tumors visible on MRI was shown to improve disease-free survival and regional/distant metastasis-free survival for patients with prostate cancer—without increasing toxicity. The aim of this study was to assess how widely this technique is being applied in current practice, as well as physicians’ perceived barriers toward its implementation.

**Methods:**

We invited radiation oncologists to complete an online questionnaire assessing their use of intraprostatic focal boost in December 2022 and February 2023. To include perspectives from a broad range of practice settings, the invitation was distributed to radiation oncologists worldwide via email list, group text platform, and social media.

**Results:**

263 radiation oncologist participants responded. The highest-represented countries were the United States (42%), Mexico (13%), and the United Kingdom (8%). The majority of participants worked at an academic medical center (52%) and considered their practice to be at least partially genitourinary (GU)-subspecialized (74%). Overall, 43% of participants reported routinely using intraprostatic focal boost. Complete GU-subspecialists were more likely to implement focal boost, with 61% reporting routine use. In both high-income and low-to-middle-income countries, less than half of participants routinely use focal boost. The most cited barriers were concerns about registration accuracy between MRI and CT (37%), concerns about risk of additional toxicity (35%), and challenges to accessing high-quality MRI (29%).

**Conclusions:**

Two years following publication of a randomized trial of patient benefit without increased toxicity, almost half of the radiation oncologists surveyed are now routinely offering focal RT boost. Further adoption of this technique might be aided by increased access to high-quality MRI, better registration algorithms of MRI to CT simulation images, physician education on benefit-to-harm ratio, and training on contouring prostate lesions on MRI.

**Supplementary Information:**

The online version contains supplementary material available at 10.1186/s13014-023-02375-5.

## Introduction

A phase III randomized controlled trial (Focal Lesion Ablative Microboost in Prostate Cancer, or FLAME) demonstrated that a focal radiotherapy (RT) boost to tumors visible on MRI improves outcomes for patients with intermediate- and high-risk prostate cancer [1]. Participants were randomly assigned to receive either uniform RT dose to the entire prostate (control arm) or RT to the entire prostate with a focal RT dose boost to gross disease (focal boost arm). Compared to the control arm, participants in the focal boost arm had improved disease-free survival, improved local control, and improved regional/distant metastasis-free survival [1, 2]. No difference in toxicity was observed between the two groups [1]. Thus, there is level 1 evidence that a meaningful oncologic benefit can be offered patients with prostate cancer without increased side effects. This approach can be delivered on RT equipment already widely available for clinical use. Two years after publication of the FLAME trial results, we sought to learn whether patients are currently able to access this benefit.

Differential adoption of focal boost may have introduced a new healthcare disparity for patients with prostate cancer. Information about radiation dose and use of focal RT boost is not routinely or publicly available. Patients may not be aware of whether they are receiving focal boost or whether this approach was even considered for them. We decided to ask radiation oncologists if they have adopted the focal boost approach. If some oncologists are offering focal boost and others are not, this would clearly imply a disparity in practice that has been shown to affect outcomes. We also asked respondents about perceived barriers to implementation of focal boost in their own practice.

## Methods

In December 2022 and February 2023, we invited radiation oncologists to complete an online questionnaire regarding focal radiotherapy boost for prostate cancer. In designing the study, we recognized two challenges. First, we are not aware of a global list of all radiation oncologists, which would be required for a survey of the complete population or to identify a random sample of that population. Second, even if all radiation oncologists could be contacted, it is likely only a small percentage would choose to participate, making accurate generalization of results impossible. Thus, robust generalizability may not be feasible. Still, a large number of responses from a diverse group of participants can be informative about practice patterns and physician perspectives. We opted for a pragmatic approach: a group of authors from varied practice settings (country of practice; academic or private; urban, suburban, or rural) used a range of electronic media to invite radiation oncologists to complete the questionnaire. While this approach would not allow formal generalization of results or calculation of a response rate (as the number of radiation oncologists contacted via social media is not known), we would be able to cast a wide net and obtain enough responses to meet the primary study goals: (1) determine whether a substantial group of radiation oncologists exists that has not already adopted focal boost for prostate cancer, and (2) gain some insight into perceived barriers to adoption.

The study was approved by the Institutional Review Board. Participants gave consent electronically. The questionnaire was designed to be very brief to encourage participation (no more than 10 min, with initial feedback suggesting typical completion in less than 3 min).

We advertised the study to our respective contacts via email and social media. We also used a previously curated email list of 850 members of the American Society for Radiation Oncology (ASTRO) practicing in the New England region of the United States and a group text-message platform for radiation oncologist members of Sociedad Mexicana de Radioterapeutas (SOMERA) with 291 users. Participants were asked to consent electronically; they self-reported as practicing radiation oncologists who treat patients with prostate cancer.

The questionnaire included 12 items (Additional file 1). We asked participants whether they use focal boost and for how many cases they typically use it per month. We also asked how often they incorporate MRI into treatment planning for prostate cancer, how many prostate cancer cases they treat in a typical month, and the degree to which their practice was genitourinary (GU)-subspecialized. We asked about fractionation schemes employed when using focal boost, how often radiologists help identify prostate tumors on imaging for treatment planning, barriers to implementing focal boost more often in their practice, and demographic information, including practice setting and years of radiation oncology experience. Finally, we conducted subgroup analyses for respondents from high-income or low-to-middle-income countries, as defined by the World Bank [3].

## Results

**Fig. 1:**
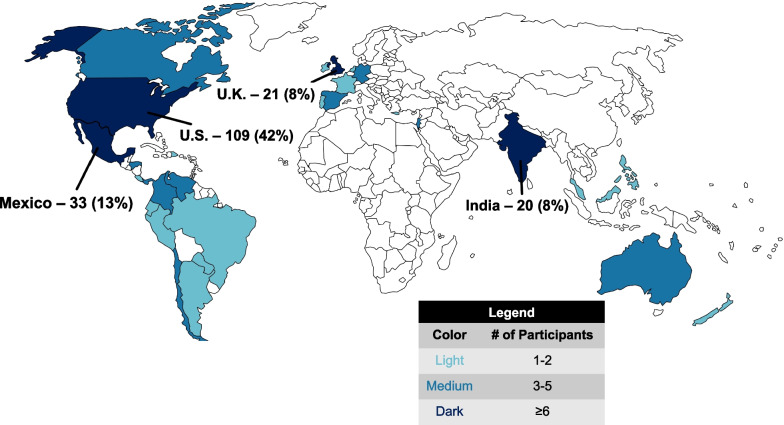
Countries represented by participants

**Fig. 2:**
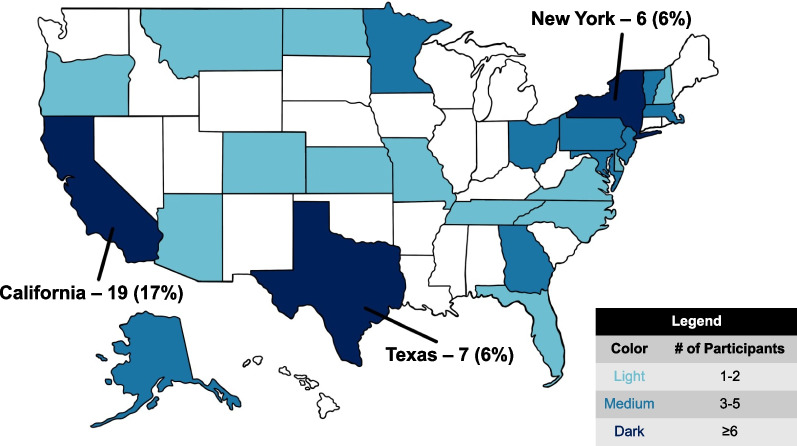
States represented by participants practicing within the United States

A total of 205 responses were initially collected over a 2-week period in December 2022 (12/6/2022–12/20/2022). Due to a low representation of generalists, the questionnaire was then reopened for 5 days in February 2023 (2/1/2023–2/6/2023) with social media posts requesting more participation from generalists, leading to a total of 263 responses. Those who reported treating zero prostate cancer cases in a typical month were then removed from the study, which lowered the total to 258 responses. The countries and states (for those in the United States) represented by participants are depicted in Figs. 1 and 2. The highest-represented countries were the United States (42%), Mexico (13%), and the United Kingdom (8%). The majority of respondents (74%) considered their practice to be at least partially GU-focused: 27% completely or nearly completely GU-focused (called hereafter “subspecialists”), 47% partially GU-focused (“partial subspecialists”), and 26% not GU-focused (“generalists”). Additional participant characteristics are provided in Additional file 1: Table S1.

**Fig. 3:**
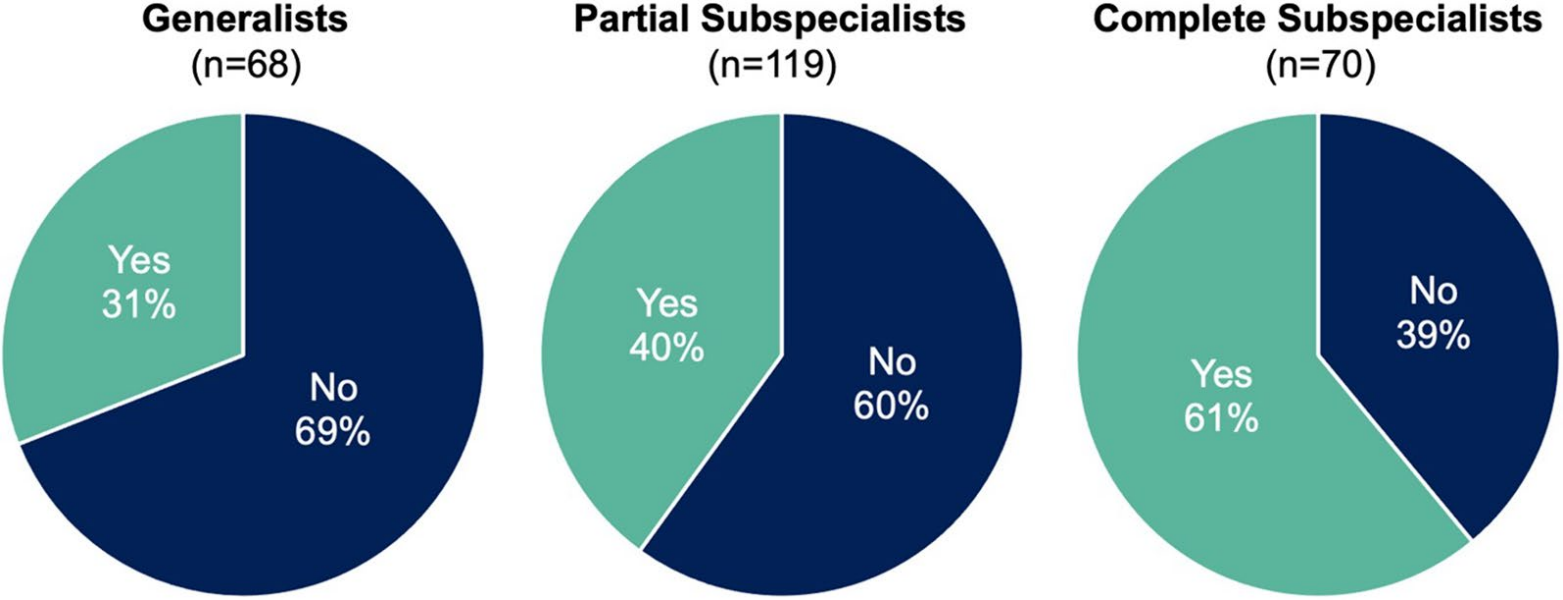
Percentages of participants who routinely use intraprostatic focal boost (“Yes”), by degree of genitourinary (GU)-subspecialization

**Fig. 4:**
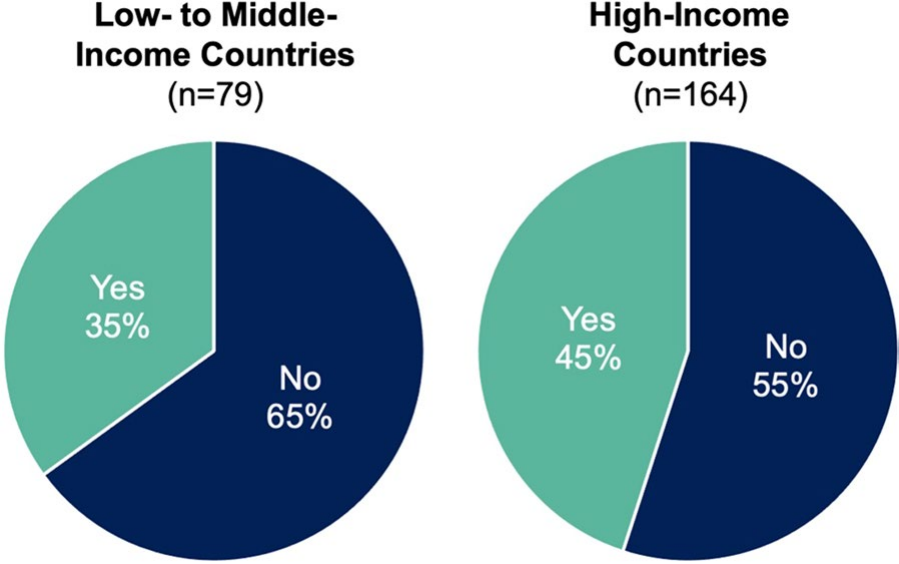
Percentages of participants who routinely use intraprostatic focal boost (“Yes”), by country’s income

Overall, 43% of participants routinely use focal boost (Table 1). Among complete subspecialists, a higher proportion (61%) routinely use focal boost (Fig. 3). Less than half of generalists and partial subspecialists, respectively, report routinely using focal boost. Likewise, less than half of participants in both high-income and low-to-middle-income countries routinely use focal boost (Fig. 4). Additional results are shown in Table 1.

**Table 1:** Reported use of intraprostatic focal radiotherapy boost, by category

Category	Routinely use focal boost (95% CI)	*p* value
Degree of GU-subspecialization		
Generalist (n = 68)	31% (21%, 43%)	0.0001
Partly subspecialized (n = 119)	40% (32%, 49%)	0.002
Completely subspecialized (n = 70)	61% (50%, 73%)	Reference
Country’s income*		
Low- to middle-income (n = 79)	35% (25%, 47%)	0.08
High-income (n = 164)	45% (37%, 53%)	Reference
Declined to state (n = 15)	67% (40%, 87%)	0.05
Practice setting		
Academic medical center (n = 133)	48% (40%, 56%)	Reference
Non-academic hospital (n = 21)	38% (19%, 62%)	0.19
Academic-affiliated community hospital (n = 38)	37% (21%, 53%)	0.10
Non-academic community hospital (n = 20)	40% (20%, 60%)	0.24
Independent/private practice (n = 44)	36% (23%, 50%)	0.08
# of PCa cases treated per month		
1–4 cases (n = 85)	34% (25%, 45%)	Reference
5–10 cases (n = 97)	41% (32%, 52%)	0.16
> 10 cases (n = 73)	55% (44%, 66%)	0.004

*GU* genitourinary, *PCa* prostate cancer

*As defined by the World Bank [3]

Study participants’ perceived barriers to implementation are shown in Table 2. Write-in answers for other barriers included: not yet part of department protocol; awaiting confirmation of safety and benefit in clinical trials; too large of a tumor or absence of a clear dominant nodule on MRI; lack of standards for lesion delineation; need to justify additional workload of boost planning to physics team; and lack of access to intensity-modulated radiation therapy (IMRT) or intrafraction motion management. Overall, the most commonly cited barriers were concerns about registration accuracy between MRI and CT (37%) and concerns about risk of additional toxicity (35%). Challenges to accessing high-quality MRI were more commonly cited by generalists (32%) and partial subspecialists (34%) compared to complete subspecialists (19%). Generalists more commonly cited lack of training on how to identify prostate tumors on MRI (22%) as a barrier.

**Table 2:** Perceived barriers to implementing intraprostatic focal boost in participants’ respective practices

Barrier	All participants (n = 258) (%)	Generalists (n = 68) (%)	Partial subspecialists (n = 119) (%)	Complete subspecialists (n = 70) (%)
Concerns about registration accuracy between MRI and CT	37	37	41	31
Concerns about risk of additional toxicity	35	34	39	27
Challenges to accessing high-quality MRI	29	32	34	19
Not aware or convinced of benefit	24	25	25	21
Have not been trained to identify prostate tumors on MRI	16	22	16	9
Dosimetrists need additional training to make high-quality plans	16	21	13	16
Prefer brachytherapy boost	14	9	13	20
Concerns about planning efficiency	10	15	9	9
Other	15	15	12	21

Participants were asked to select all that apply

Among those offering focal RT boost, participants reported using a range of fractionation schemes when including a boost (Table 3). The most common fractionation scheme overall was moderate hypofractionation (2.1–3 Gy/fraction to the whole prostate) (70%), followed by ultrahypofractionation (≥ 6 Gy/fraction to the whole prostate) (45%). Generalists favored using standard fractionation when delivering a focal RT boost more than subspecialists did, with 43% of generalists using this scheme compared to 25% of subspecialists. 2% of participants selected “Other” and elaborated that they used brachytherapy boost.

**Table 3:** Reported fractionation schemes among participants who routinely use intraprostatic focal boost

Fractionation scheme	All participants (n = 112) (%)	Generalists (n = 21) (%)	Partial subspecialists (n = 48) (%)	Complete subspecialists (n = 43) (%)
Standard fractionation (1.8–2 Gy/fraction to the whole prostate)	29	43	31	19
Moderate hypofractionation (2.1–3 Gy/fraction to the whole prostate)	70	62	69	74
Ultrahypofractionation (≥ 6 Gy/fraction to the whole prostate)	45	24	38	63
Other	2	0	0	5

Respondents were asked to select all that apply

## Discussion

Two years after publication of level 1 evidence supporting focal RT boost, almost half of radiation oncologist respondents in our study have adopted this approach for their patients with prostate cancer. Subspecialists whose clinical practice focuses completely or nearly completely on genitourinary cancers were more likely to report use of focal boost. Nonetheless, a large proportion (39%) of these experts is not routinely using focal boost. Our results show a healthcare disparity exists where only patients seeing certain physicians will even be considered for focal boost.

It is important to note that rapid adoption of a new radiotherapy approach is not common, and widespread implementation of proven benefits is expected to take time. For example, despite multiple randomized clinical trials demonstrating noninferiority of moderate hypofractionation compared to conventional fractionation in prostate cancer treatment, adoption of hypofractionated regimens has been slow and variable across healthcare settings and individual physicians [4–6]. Such has been the case for other disease sites such as breast cancer, where moderate hypofractionation in adjuvant radiotherapy has been gradually integrated over nearly two decades [7, 8]. It is thus notable that a substantial proportion of the radiation oncologists surveyed are already routinely offering focal boost to their patients. Furthermore, there are certain barriers that have likely contributed to the slow uptake of hypofractionation, including a major financial disincentive induced by a fee-for-service payment model and concerns regarding the potential for increased toxicity [9, 10]. In contrast, the financial impact of the focal boost approach is less clear, and it has been shown to improve cancer outcomes without increasing toxicity.

Participants provided critical insight into barriers to their increased use of focal boost. Efforts to improve patient outcomes might address the most frequently cited barriers to adoption, including lack of access to high-quality MRI and concerns about accuracy of registration between MRI and CT images. Lack of access to high-quality MRI was more common in low-to-middle-income countries but remained a commonly cited barrier in high-income countries as well. Accurate registration between prostate MRI and CT is challenging due to variations in prostate appearance and pelvic anatomy, which can be attributed in part to movement of the prostate between scans [11]. While registration of the overall pelvis and pelvic bones is relatively straightforward, for focal boost, the goal is to register the prostate, which is difficult to precisely identify on CT and whose position is affected by variable bladder and rectal filling. Several methods have been developed to improve registration, including the use of implanted fiducials within the prostate, which were used in the FLAME trial [12]. Numerous automatic registration tools have also been developed that utilize machine learning and may aid in accurate and efficient registration of the prostate between MRI and planning CT [13–15].

Another commonly perceived barrier, especially among generalists, is the lack of training to identify prostate tumors on MRI. Lesion identification is not a common component of radiation oncology training. In the FLAME trial, oncologists had the assistance of expert radiologists for each case. Improved technology can also help to overcome this barrier. For example, a novel prostate cancer MRI biomarker (called the Restriction Spectrum Imaging restriction score, or RSI_rs_) makes it easier to see clinically significant cancer [16–19]. RSI_rs_ can be obtained on clinical scanners with a 2–4-min diffusion-weighted acquisition, in addition to anatomic *T*_*2*_-weighted MRI. In a prospective study, use of RSI_rs_ markedly improved radiation oncologists’ accuracy in identifying prostate tumors [20].

Some participants expressed doubt about the benefit of focal boost, despite the results of the FLAME trial. The initial FLAME paper reported only a disease-free survival advantage of focal boost, with an increase from 85 to 92% at 5 years compared to the standard arm [1]. Some physicians may be unaware of the regional/distant metastasis-free survival advantage described in a subsequent publication, where the regional and distant metastatic failure rate was reduced by nearly half in the focal boost arm [2]. Additional ongoing trials may also corroborate the FLAME results, encouraging adoption.

Other participants expressed valid concerns about the potential for increased toxicity. In the FLAME trial, the cumulative incidence of late grade ≥ 2 GU toxicity was 23% in the standard arm and 28% in the focal boost arm, whereas that of late grade late grade ≥ 2 GI toxicity was 12% in the standard arm and 13% in the focal boost arm [1]. These differences were small and not statistically significant. The focal boost dose on that trial was escalated up to 95 Gy but only to the extent feasible while meeting dose constraints to normal tissues, suggesting that prioritizing organ at risk constraints allows focal boost doses to be delivered safely yet effectively.

Additionally, some participants had concerns about focal boost in the setting of larger doses per fraction than used in FLAME. Data on this topic are emerging [21]. Hypo-FLAME was a phase II, single-arm study of ultra-hypofractionation (5 weekly fractions) with a focal boost up to 50 Gy. This study incorporated a urethral dose constraint, as recommended by Groen et al., and found acceptable toxicity [22]. Phase III trial evidence is not available for focal boost with ultra-hypofractionated regimens. DELINEATE was a single-center phase II trial in the UK that recently demonstrated safety and feasibility of using a focal boost of 67 Gy in 20 fractions or 82 Gy in 37 fractions. The efficacy and toxicity rates at 5 years were comparable to those in published trials, including FLAME. PIVOTALboost is an ongoing phase III randomized trial in the UK evaluating a focal boost of 67 Gy in a 20-fraction hypofractionated regimen [23]. Ideal constraints are still under investigation, and some patients may not be good candidates for boosting [24]. On the other hand, if hypofractionation is considered a key barrier to boosting, the logistic advantages of hypofractionation must be weighed against the oncologic benefit of focal boost.

Although not directly addressed in our questionnaire, implementation strategies are also important in increasing adoption of new techniques. Implementation science is the study of methods that seek to promote the uptake of evidence-based practices. System-wide changes rely on structural and organizational support to enable the initiation and expansion of implementation strategies. This includes clinical networks, which are comprised of healthcare leaders who aim to identify areas of improvement in clinical care and service delivery and advocate for system-wide change using a collaborative approach [25]. Clinician-centered educational programs targeting behavioral change have the potential to improve guideline adherence at a regional level. For example, one such educational program reduced unnecessary imaging in men with localized, low-risk prostate cancer [26]. At the provider level, the clinical champion approach is especially effective at impacting individual physician behavior. Strategies like these aim to accelerate the integration of research findings into clinical practice by identifying and addressing contextual barriers that contribute to the evidence-practice gap [27].

The FLAME trial applied standard clinical techniques in widespread use today. However, additional technologies may play a role in expanding the feasibility of focal RT boost. For example, a posterior tumor may not be amenable to a robust focal tumor boost without violating rectal dose constraints, but placement of a hydrogel spacer could yield more favorable dosimetry for the focal boost. Similarly, adaptive planning and MR-linac platforms could facilitate tighter planning margins and/or more accurate focal boosting. Focal-only brachytherapy boost and intensity modulated proton therapy boost are also possible areas for further study.

Limitations of this study include self-reported practice patterns and a sample of convenience, which led to the overrepresentation of physicians at academic medical centers and of genitourinary subspecialists. The questionnaire was also very brief to encourage participation and does not provide a comprehensive picture of all aspects of practice patterns, including how physicians who do offer focal boost select candidates for this approach or how they identify the target volumes.

In conclusion, in responses from over 250 international radiation oncologists, we found that almost half are routinely offering focal RT boost. Of note, there was overrepresentation in our study of subspecialists in genitourinary cancers, who might be earlier adopters. Based on commonly cited barriers, further adoption of focal RT boost might be aided by increased access to high-quality MRI, better registration algorithms of MRI to CT simulation images, more clinical data (especially for larger fraction sizes), physician education on benefit-to-harm ratio, and physician training on how to contour prostate lesions on MRI. Addressing these barriers would likely increase the adoption of focal RT boost and improve the efficacy of RT for more patients with prostate cancer.

### Supplementary Information

**Additional file 1: Table S1.** Participant characteristics.

## Data Availability

De-identified data are available to bona fide researchers for non-commercial use upon request.

## Abbreviations

FLAME: Focal Lesion Ablative Microboost in Prostate Cancer
RT: Radiotherapy
GU: Genitourinary
RSIrs: Restriction spectrum imaging restriction score
