# Supplementary material for: Use of focal radiotherapy boost for prostate cancer: radiation oncologists’ perspectives and perceived barriers to implementation
Source: Radiat Oncol. 2023 Nov 11;18:188. doi: 10.1186/s13014-023-02375-5 (PMC10638743; doi:10.1186/s13014-023-02375-5)
Supplement: Supplementary file 1 — Additional file 1: Table S1. Participant characteristics. [file 13014_2023_2375_MOESM1_ESM.docx]

**Additional file 1: Table S1.** Participant characteristics.

| **Country/nation of practice** | **n (%)** |
| --- | --- |
| United States of America | 109 (42%) |
| Mexico | 33 (13%) |
| United Kingdom | 21 (8%) |
| India | 20 (8%) |
| Australia | 5 (2%) |
| Chile | 5 (2%) |
| Colombia | 5 (2%) |
| Spain | 5 (2%) |
| Honduras | 4 (2%) |
| Venezuela | 4 (2%) |
| Canada | 3 (1%) |
| Germany | 3 (1%) |
| Israel | 3 (1%) |
| Other (≤2 respondents per country/nation) | 23 (9%) |
| Declined to state | 15 (6%) |
| **State of practice (109 US respondents only)** | **n (%)** |
| California | 19 (17%) |
| Texas | 7 (6%) |
| New York | 6 (6%) |
| Minnesota | 5 (5%) |
| Pennsylvania | 5 (5%) |
| Massachusetts | 4 (4%) |
| Alaska | 3 (3%) |
| Georgia | 3 (3%) |
| Maryland | 3 (3%) |
| New Jersey | 3 (3%) |
| Ohio | 3 (3%) |
| Vermont | 3 (3%) |
| Other (≤2 respondents per state) | 17 (16%) |
| Declined to state | 28 (26%) |
| **Practice setting** | **n (%)** |
| Academic medical center | 133 (52%) |
| Non-academic hospital | 21 (8%) |
| Academic-affiliated community hospital | 38 (15%) |
| Non-academic community hospital | 20 (8%) |
| Independent/private practice | 44 (17%) |
| **Practice community** | **n (%)** |
| Urban | 180 (70%) |
| Suburban | 61 (24%) |
| Rural | 16 (6%) |
| **Years in practice since completing training** | **n (%)** |
| Still in training | 10 (4%) |
| <5 years | 62 (24%) |
| 5-10 years | 61 (24%) |
| >10 years | 125 (48%) |
